# Supplementary figures and images for: A Whole-Genome Assembly for Hyaloperonospora parasitica, A Pathogen Causing Downy Mildew in Cabbage (Brassica oleracea var. capitata L.)
Source: J Fungi (Basel). 2023 Aug 3;9(8):819. doi: 10.3390/jof9080819 (PMC10456066; doi:10.3390/jof9080819)

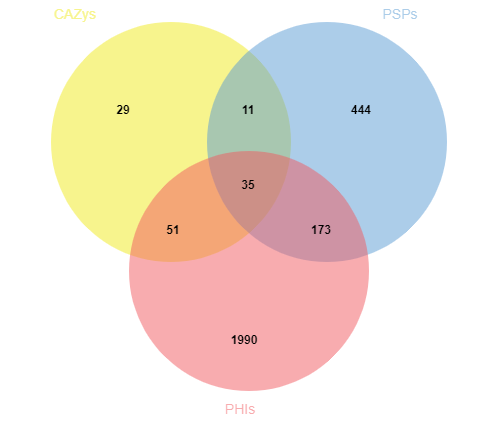

Supplement: Supplementary file 1 [file jof-09-00819-s001.zip › Supplement Figure S2 Relationships between genes in three annotated plates.png]

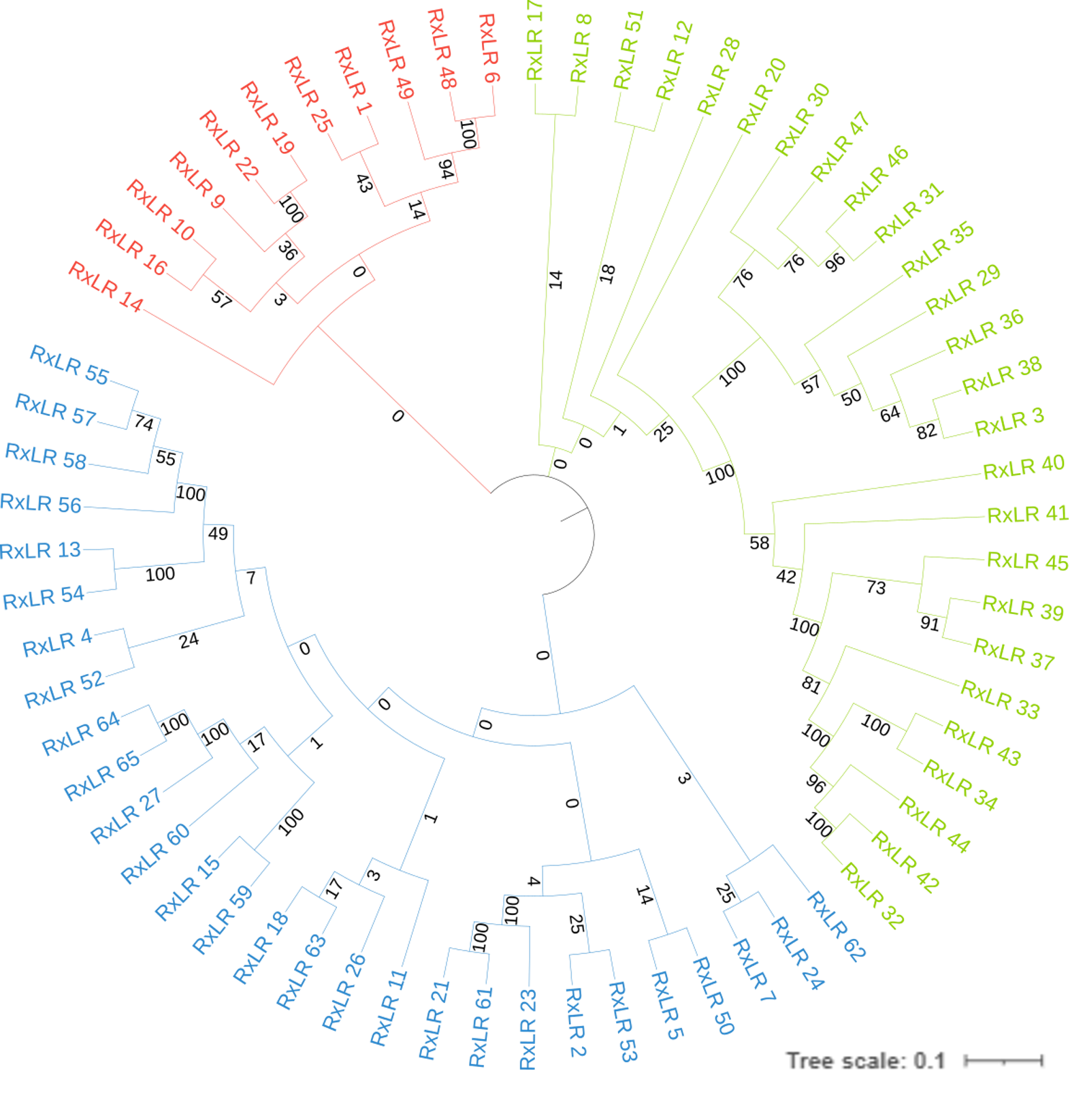

Supplement: Supplementary file 1 [file jof-09-00819-s001.zip › Supplement Figure S3 Phylogenomic analysis among 65 RxLR effectors.png]

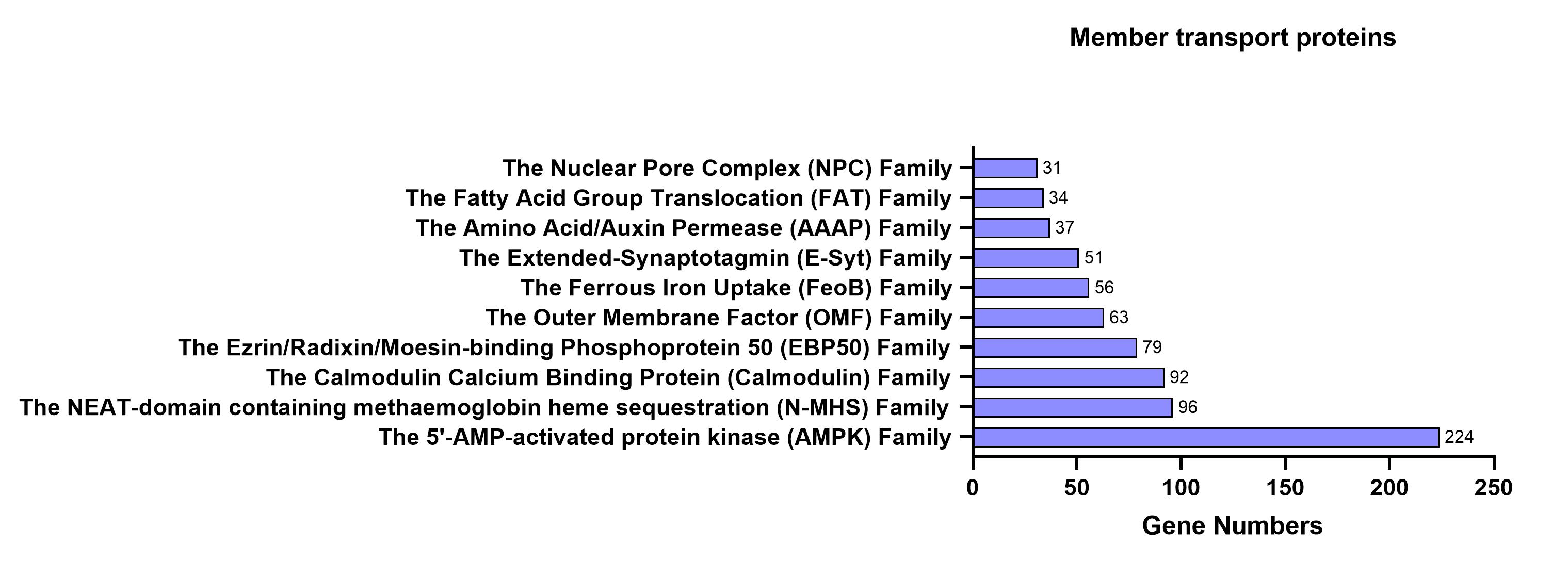

Supplement: Supplementary file 1 [file jof-09-00819-s001.zip › Supplement Fiugre S1 Member transport protrins.jpg]
